# Risks of electromagnetic fields from the perspective of general practitioners and pediatricians

**DOI:** 10.1186/s12875-025-02762-9

**Published:** 2025-03-03

**Authors:** Felix Forster, Claudia Riesmeyer, Lyn Ermel, Katharina Lüthy, Ronny Jung, Tobias Weinmann

**Affiliations:** 1 Institute and Clinic for Occupational, Social and Environmental Medicine, LMU University Hospital, LMU Munich, Ziemssenstr. 5, 80336 München, Germany; 2 Department of Media and Communication, LMU Munich, Oettingenstr. 67, 80538 München, Germany; 3 Berufsverband der Kinder- und Jugendärzte (BVKJ), Mielenforster Str. 2, 51069 Köln, Germany

**Keywords:** Electromagnetic fields, General practitioners, Paediatricians, Risk assessment

## Abstract

**Background:**

Although there is little evidence for adverse health effects due to exposure to electromagnetic fields (EMF) below legal limits, worries regarding these effects are relatively frequent in the general population. For many individuals, general practitioners (GPs) and pediatricians are the first point of contact with the health system. Therefore, it is essential to understand their EMF risk perception.

**Methods:**

We conducted a cross-sectional mixed methods study inviting 3,000 GPs and 2,000 pediatricians sampled from the German Federal Medical Registry, of which 614 (12.3%) participated in an online survey and 25 participated in focus groups. We estimated the prevalence of high risk perception, poor subjective knowledge regarding EMF, and the relevance of EMF in their everyday work correcting for non-response by Multilevel Regression and Poststratification.

**Results:**

About a quarter of physicians indicated high risk perception regarding health and EMF. Relevance was low, with about 40% of GPs and about 20% of pediatricians reporting EMF-related consultations during the last year. About 60% of physicians had poor subjective knowledge. Many physicians said they could not rule out the possibility of adverse health effects of EMF due to insufficient knowledge and expressed a need for information to address this knowledge gap.

**Conclusions:**

A substantial part of GPs and pediatricians with high risk perception are physicians with poor subjective knowledge regarding EMF who cannot completely rule out EMF below legal limits as a cause of unspecific, unclear symptoms, and who are therefore open to patients’ suggestions of EMF as a potential cause.

**Supplementary Information:**

The online version contains supplementary material available at 10.1186/s12875-025-02762-9.

## Background

The electromagnetic spectrum ranges from static and extremely low-frequency electric and magnetic fields through radiofrequency electromagnetic fields, infrared radiation, and visible light, up to ultraviolet, x-ray, and gamma radiation. The upper end of the electromagnetic spectrum has enough energy to ionize and thereby affect the atomic structure, while frequencies below ultraviolet radiation are non-ionizing. For the presented study, we consider risk perception regarding the lower end of the electromagnetic spectrum ranging from static up to radiofrequency electromagnetic fields (0 Hz-300 GHz), hereafter called electromagnetic fields (EMF) [1, 2]. Since EMF carry less energy than visible or infrared light, they are as well classified as non-ionizing radiation. Nonetheless, EMF can have biological effects like nerve stimulation (mainly low-frequency EMF up to 10 MHz), heating effects (mainly radiofrequency EMF above 100 kHz), and changes in the permeability of cell membranes (mainly pulsed low-frequency EMF) [2]. In order to protect the population from these potentially adverse effects, legal limits have been set which, in Germany, are based on recommendations by the International Commission on Non-Ionizing Radiation Protection (ICNIRP) and the German Commission on Radiological Protection. ICNIRP recommends limits at which the biological effects mentioned above do not lead to adverse health effects [1, 2]. In addition, studies were conducted investigating other health outcomes, most of which do not seem to be caused by EMF below legal limits, e.g., brain tumors [3, 4] or non-specific symptoms [5], and some of which need additional investigation, e.g., the potential association of extremely low-frequency magnetic fields and childhood leukemia [6, 7], as well as radiofrequency EMF and certain aspects of brain activity [8–10]. Concerning an outcome such as cognitive performance, there is some degree of uncertainty as well [11, 12]. A specific challenge in this context may be to disentangle potential biophysical effects (i.e., effects directly related to sources of EMF) from behavioral / psychological effects or mechanisms (e.g., reduced sleep quality due to excessive smartphone use) [5]. The latter may be especially relevant for some endpoints that specifically apply to children and adolescents such as symptoms of attention deficit and hyperactivity. The fact that for some endpoints, and also for the question if children and adolescents are particularly vulnerable to EMF exposure, the existing body of evidence does not allow to draw final conclusions, may be one of the reasons that potential health effects of EMF are a topic that is frequently subjected to controversial debate [13]. 

Accordingly, worries regarding adverse health effects due to EMF exposure below legal limits are relatively frequent in the general population. In a survey among the German general population, 62% of the participants indicated that in their view they have a lot of contact to ‘radiation from cell phones’ with only 30% feeling that it is possible to adequately protect from this exposure [14]. These worries and uncertainties can lead to suspicions regarding EMF as a potential cause of health conditions and a need for clarification of these suspicions. In this context, general practitioners (GPs) and pediatricians are among the first points of contact in the health system for most individuals, giving them an important role in the dissemination of information among the general population. In previous studies, GPs also reported considerable concern regarding potential adverse health effects of EMF, as well as insufficient knowledge regarding EMF [15–19]. However, a substantial part of the evidence regarding health effects of EMF was published after many of these surveys have been conducted, e.g., results from the MOBI-Kids study [4], and patterns of media use changed during the last 10–15 years [20]. To what extent these developments have influenced physicians’ risk perception concerning EMF is unknown. In addition, it is mostly unclear by which factors risk perception and knowledge regarding EMF, as well as relevance of EMF in physicians’ everyday work are determined. New viewpoints like a communication science perspective and qualitative research methods might help improve understanding as they allow to elucidate attitudes and opinions in greater depth because in qualitative studies it is easier for participants to articulate habitual actions and to express complex and unconscious perceptions. Quantitative and qualitative results can thus together provide a clearer and more comprehensible picture of the views of GPs and pediatricians on the risks of EMF than surveys alone. Furthermore, no study so far has scrutinized pediatricians’ view on EMF and health. Their perspective is particularly interesting though since children could be a vulnerable group regarding adverse health effects of EMF [6] and regarding their use of communication technologies [21].

This study thus investigated risk perception regarding EMF, relevance of EMF in everyday work, and subjective knowledge regarding EMF in GPs and pediatricians. We conducted a cross-sectional online survey and combined it with qualitative methods in a mixed methods approach to additionally consider a communication science perspective.

## Methods

We conducted a mixed methods study using a sequential explanatory design [22, 23], i.e., starting with a quantitative online survey and continuing with qualitative guideline-based focus groups in a subset of online survey participants with the goal of further contextualizing the survey results. The quantitative and qualitative sub-studies were analyzed separately at first before their results and conclusions were subsequently combined for deeper understanding. Data collection was performed between February and August 2023.

This study was performed in compliance with relevant laws and institutional guidelines as well as the Declaration of Helsinki and was approved by the Ethics Committee at the Medical Faculty of Ludwig-Maximilians-University (LMU) Munich, Germany (20th Sep 2022). Written informed consent was obtained from all participants for the survey (including for linking questionnaire and registry data) and focus groups.

### Sample

From the German Federal Medical Registry, 3,000 GPs and 2,000 pediatricians were sampled using stratified random sampling by federal state and additional training in homeopathy (see section “Additional variables” below). The Federal Medical Registry contains all statutory health insurance physicians and psychotherapists. Therefore, physicians in hospitals and physicians who only get paid by patients directly or private insurance were not part of the sample. Sampling was performed by the National Association of Statutory Health Insurance Physicians, who hold the data, in January 2023 after approval by the Federal Ministry of Health. All 5,000 physicians were invited by postal letters to fill in an online questionnaire. They received up to two reminders including a short hardcopy version of the questionnaire, which only comprised questions regarding the outcome variables. The quantitative online survey was conducted using the online questionnaire tool LimeSurvey on internal servers of the LMU University Hospital.

### Outcomes

Three outcomes were investigated: risk perception regarding EMF, relevance of EMF in everyday work, and subjective knowledge regarding EMF. The questionnaire was, in part, based on previous work [15, 16] and compiled for the presented study (see Additional file 1). Risk perception was measured by specifying the degree of agreement with two statements on a 5-point Likert scale: “There are individuals who develop adverse health effects from electromagnetic fields below legal limits” (hereafter: EMF effects below legal limits) and “Adverse health effects from electromagnetic fields are mainly psychosomatic” (hereafter: EMF effects are psychosomatic). For data analysis, both items were dichotomized (agreement vs. no agreement), with the center category being defined as “no agreement”. In addition, physicians were asked multiple-choice questions about which adverse health effects they think can be caused by EMF (hereafter: physician-rated health effects) and which EMF sources produce fields that can cause them (hereafter: physician-rated EMF sources). Relevance of EMF in everyday work was measured by asking how often physicians have been consulted regarding EMF during the last 12 months (never, 1–4 times, 5–9 times, 10–49 times, 50–99 times, ≥ 100 times). This item was dichotomized as well (ever vs. never). Subjective knowledge regarding EMF was measured by rating on a 5-point Likert scale how well-informed they perceive themselves regarding potential adverse health effects of EMF. This item was dichotomized (poorly informed vs. well informed), with the center category being evaluated as “well informed” because feeling poorly informed was the outcome of interest. In addition, a multiple-choice question evaluated which sources physicians use when searching for information on adverse health effects of EMF (hereafter: information sources).

### Additional variables

Data from the Federal Medical Registry included socio-demographic characteristics, namely sex (male, female), age group (≤ 40 years, 41–50 years, 51–60 years, > 60 years), federal state (all 16 German states) and type of municipality in which the medical practice is located (large town: ≥ 100,000 inhabitants; medium town: < 100,000 & ≥ 20,000; small town: < 20,000 & ≥ 10,000; village: < 10,000 & ≥ 5,000; rural municipality: < 5,000; [24]), type of physician (GP, pediatrician), and additional training in alternative medicine (TAM). Due to previous results indicating that the type of TAM is relevant for physicians’ risk perception towards EMF [25], we considered three different definitions of TAM: additional training in homeopathy (yes vs. no; TAM 1), additional training in homeopathy or acupuncture (yes vs. no; TAM 2), and additional training in homeopathy or acupuncture or naturopathic treatment (yes vs. no; TAM 3). Registry data was available for all participants.

### Focus groups

After completing the online questionnaire, physicians were invited to participate in focus groups. Because physicians were located all over Germany, focus groups were conducted online with 2–4 participants each (planned were 5 participants each) and with the intention to mix by state and type of municipality. Due to cancellations at short notice, some focus groups had to be conducted as in-depth interviews with a single participant. The guideline was developed based on the literature as well as the online survey results (see Additional file 2). Interviews and focus groups were recorded, transcribed, and pseudonymized.

### Data analysis

For the quantitative sub-study, socio-demographic characteristics were analyzed descriptively and compared between the study population (population that participated), the sample (population that was invited), and the source population (population from which the sample was drawn; based on aggregated data used for poststratification as described below). ‘Physician-rated health effects’, ‘physician-rated EMF sources’, and ‘information sources’ are reported descriptively as well. These three questions were not asked in the short questionnaire that was sent out with the reminders. Prevalence of the dichotomized items ‘EMF effects below legal limits’, ‘EMF effects are psychosomatic’, ‘EMF relevance’, and ‘subjective EMF knowledge’ were estimated by calculating empirical estimates and estimates corrected for non-response. Empirical point estimates were relative frequencies, while empirical interval estimates were defined as the 95% confidence intervals (CI) for proportions (see, e.g [26]). Estimates corrected for non-response were calculated using Multilevel Regression and Poststratification (MRP) [27, 28]. MRP is a two-step process: Firstly, prevalence is estimated by a Bayesian multilevel logistic regression model with correction variables as predictors. In this case, correction variables were socio-demographic variables from the Federal Medical Registry, i.e., sex, age group, state, type of municipality, type of physician, and TAM leading to 2,560 different strata, i.e., different combinations of the categories of these variables. Age group, state, and type of municipality were included into the model as varying intercepts, while the binary correction variables sex, type of physician, and TAM were included as predictors for computational efficiency [29]. Separate models were calculated for the four items. MRP was repeated three times for every item, using a different TAM definition each time. Secondly, in the poststratification step, external aggregated data on the population distribution across the 2,560 strata was used to weight the estimates for each stratum to form a single estimate for the population. The external data came from the Federal Medical Registry and corresponded to the source population. External data was also available three times, once for each TAM definition. Therefore, corrected estimates describe the outcome prevalence among all German statutory health insurance GPs and pediatricians. By only considering a subset of strata, e.g., only the 1,280 strata with GPs, a corrected sub-group prevalence can be estimated. Corrected estimates by type of physician were calculated for all four items. For item ‘EMF effects below legal limits’, corrected estimates by sex, TAM, and type of municipality were calculated as well. Individuals with missing outcome values were excluded from the corresponding models. Estimates were summarized by median (point estimate) and 95%-posterior interval (interval estimate). Data processing and quantitative analyses were conducted in R version 4.1.1 [30]. Bayesian models were calculated in Stan [31] using the rstanarm package [32]. In four chains, 2,000 samples per chain (of which 1,000 were warm-up samples) were drawn, leading to 4,000 usable samples per model. As priors, the weakly informative default priors of the rstanarm package were used. Model diagnostics were checked for any problems during sampling [33]: effective sample size, $\:\widehat{R}$, tree depth, energy, and trace plot inspection indicated no problems. A few divergent transitions occurred but could be avoided by decreasing step size (parameter adapt_delta = 0.99).

For the qualitative sub-study, pseudonymized transcripts were analyzed theory-based, building the category system on literature and survey results. Additional categories were added inductively from the transcripts. All transcripts were read multiple times and participants’ statements were sorted into (sub-)categories using MAXQDA software. The analysis aimed to identify similarities and differences between physicians to better understand their perceptions and assessments, which helps put the quantitative survey results into context.

## Results

In total, 614 (12.3% of the 5,000 invited) physicians participated in the quantitative sub-study, 292 in the online survey and 322 in the short questionnaires that were sent out with the reminders. In the source population, i.e., the registry, the number of GPs was much higher (Table 1) but we oversampled pediatricians to be able to investigate both sub-groups. Younger physicians and those with TAM were more likely to participate. For the focus groups, 25 physicians (15 GPs and 10 pediatricians) representing different German states and types of municipalities could be recruited. In total, 8 focus groups with 2–4 participants and 3 in-depth interviews with a single participant were conducted.

**Table 1:** Distribution of socio-demographic variables by population

Variable	Source population	Sample	Study population
Total	62,040 (100%)	5,000 (100%)	614 (100%)
Type of physician			
GPs	54,658 (88.1%)	3,000 (60.0%)	329 (53.6%)
Pediatricians	7,382 (11.9%)	2,000 (40.0%)	285 (46.4%)
Sex			
Male	30,520 (49.2%)	2,270 (45.4%)	296 (48.2%)
Female	31,520 (50.8%)	2,730 (54.6%)	318 (51.8%)
Age groups			
≤ 40 years	5,969 (9.6%)	525 (10.5%)	81 (13.2%)
41–50 years	14,983 (24.2%)	1,304 (26.1%)	192 (31.3%)
51–60 years	21,793 (35.1%)	1,767 (35.3%)	199 (32.4%)
> 60 years	19,295 (31.1%)	1,404 (28.1%)	142 (23.1%)
Type of municipality			
Large town	21,027 (33.9%)	1,796 (35.9%)	209 (34.0%)
Medium town	18,730 (30.2%)	1,668 (33.4%)	206 (33.6%)
Small town	9,751 (15.7%)	750 (15.0%)	94 (15.3%)
Village	7,914 (12.8%)	540 (10.8%)	62 (10.1%)
Rural municipality	4,618 (7.4%)	246 (4.9%)	43 (7.0%)
Additional training in homeopathy (TAM 1)			
Yes	1,649 (2.7%)	134 (2.7%)	24 (3.9%)
No	60,391 (97.3%)	4,866 (97.3%)	590 (96.1%)
Additional training in homeopathy or acupuncture (TAM 2)			
Yes	5,190 (8.4%)	326 (6.5%)	54 (8.8%)
No	56,850 (91.6%)	4,674 (93.5%)	560 (91.2%)
Additional training in homeopathy or acupuncture or naturopathic treatment (TAM 3)			
Yes	8,569 (13.8%)	536 (10.7%)	82 (13.4%)
No	53,471 (86.2%)	4,464 (89.3%)	532 (86.6%)

Source population: population from which the sample was drawn; Sample: population that was invited; Study population: population that participated

**Fig. 1:**
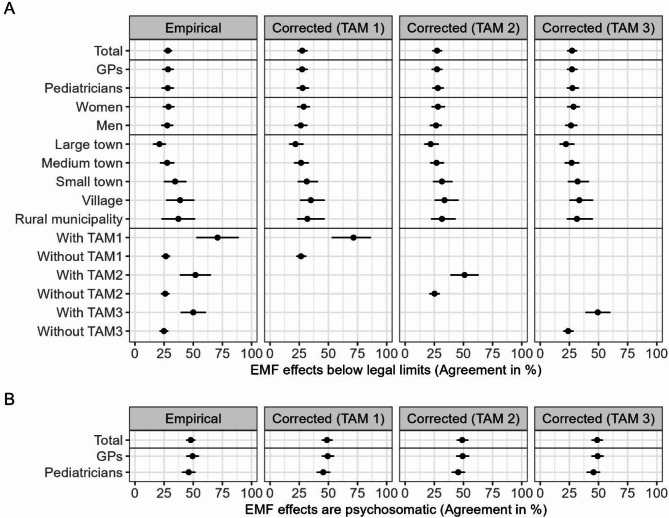
Proportion of physicians with high risk perception regarding electromagnetic fields (EMF). High risk perception was measured by agreement with two different statements (**A**: ‘EMF effects below legal limits’, **B**: ‘EMF effects are psychosomatic’) in the study population (empirical) and corrected for non-response using MRP (corrected) in three versions depending on the definition of additional training in alternative medicine (TAM); total and by type of physician, sex, type of municipality, and TAM; ‘EMF effects below legal limits’: *N* = 606, ‘EMF effects are psychosomatic’: *N* = 608, difference to total number of *N* = 614 due to missing values in the corresponding outcome

In the study population, 28.2% agreed with the statement that there are individuals who develop adverse health effects from EMF below legal limits with very similar numbers for GPs and pediatricians, as well as male and female physicians (Fig. 1.A). Corrected for non-response, the proportion of agreement was estimated to be 27.6% (95%-CI: 23.5-31.9%), using TAM definition 1 (homeopathy yes vs. no) for correction. Using a different TAM definition led to similar results. The proportion of agreement varied by type of municipality, with the highest values in villages, where an estimated 35.0% (95%-CI: 25.0-47.1%) agreed, and the lowest values in large towns, where an estimated 22.2% (95%-CI: 16.6-28.8%) agreed (both corrected using TAM 1). Among physicians with additional training in homeopathy, an estimated 71.3% (95%-CI: 52.2-85.9%) agreed, while when including acupuncture and naturopathic treatment, only around half of physicians with TAM did. In a second question addressing risk perception regarding EMF, 47.9% of participating physicians agreed that adverse health effects from EMF are mainly psychosomatic with slightly higher values in GPs (Fig. 1.B). The corrected estimates moved closer to the GPs’ values due to the higher proportion of GPs in the source population. Physician-rated health effects that were selected most were unspecific symptoms like sleep disorders (45.7%), headaches (44.1%), nervousness/restlessness (38.0%), and difficulties concentrating (37.0%), while the most frequent physician-rated EMF sources were cell phone base stations (41.6%), cell phones (40.1%), power lines (34.9%), and WIFI / Bluetooth / computers (31.4%).

In the focus groups, physicians mostly said that they consider adverse health effects of EMF below legal limits as unlikely but that they cannot rule out the possibility due to insufficient knowledge. They further said that, when EMF are suspected to cause the patients’ conditions, the patients usually bring up EMF themselves. The participants reported that these conditions are frequently characterized by unspecific, unclear symptoms that likely have multiple causes. Physicians indicated that they consider their patients’ suggestions because they want to create open communication between them and, as mentioned, cannot completely rule out EMF as a cause. One GP said: “I wouldn’t talk anybody out of it, saying ‘That makes no sense, that is impossible’. I can share the concern that somebody has, but I think it’s hard to find reliable information.” (translated from German by the authors). Nonetheless, physicians usually still try to find evidence-based causes as well.

**Fig. 2:**
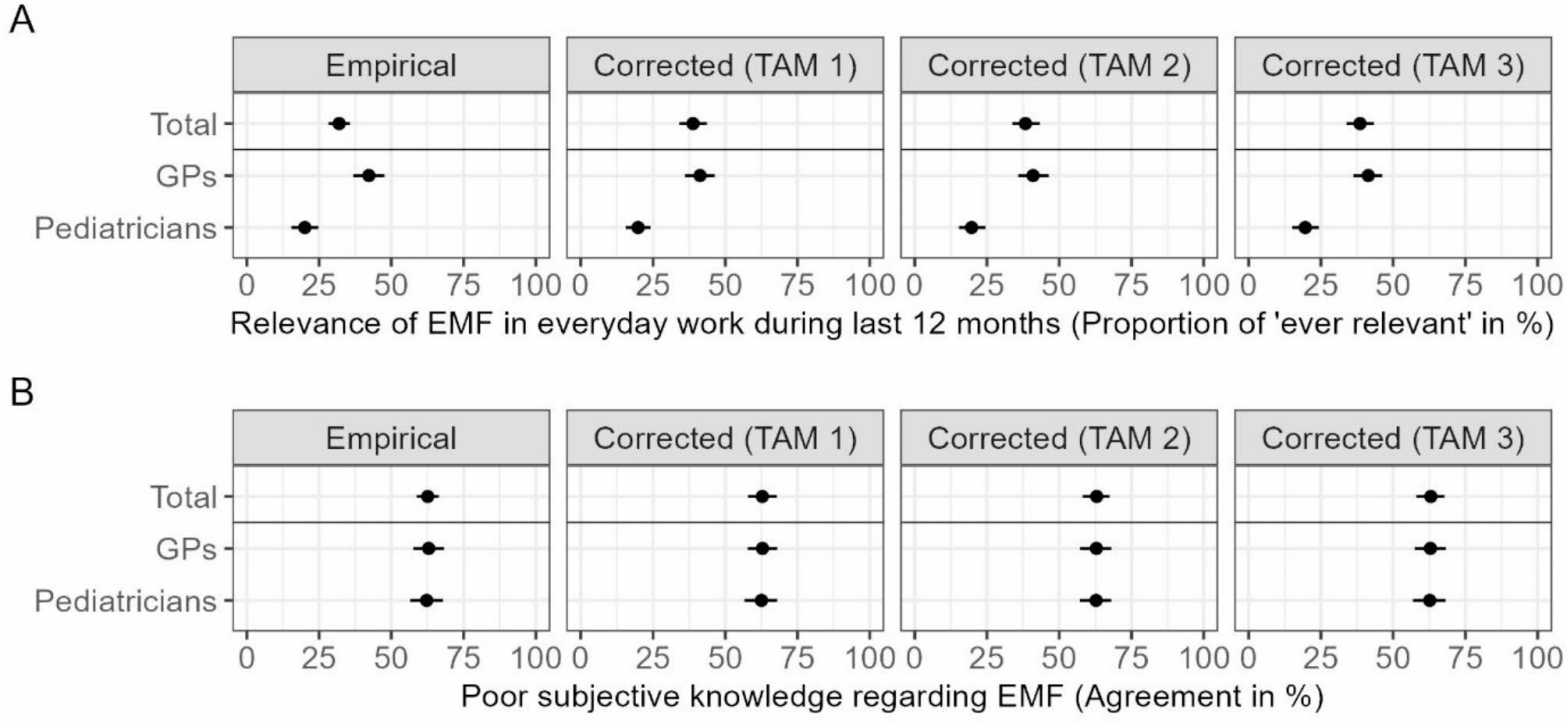
Proportion of physicians reporting relevance of electromagnetic fields (EMF) and poor subjective knowledge regarding EMF. Proportion of physicians reporting relevance of EMF in everyday work during the last 12 months (**A**) and physicians with poor subjective knowledge regarding EMF (**B**) in the study population (empirical) and corrected for non-response using MRP (corrected) in three versions depending on the definition of additional training in alternative medicine (TAM); total and by type of physician; ‘Relevance of EMF’: *N* = 601, ‘poor subjective knowledge regarding EMF’: *N* = 599, difference to total number of *N* = 614 due to missing values in the corresponding outcome

The quantitative sub-study found that EMF relevance in physicians’ everyday work is generally low with large differences between GPs and pediatricians (Fig. 2.A). For GPs, an estimated 41.5% (95%-CI: 36.3-47.0%) were consulted regarding EMF during the last 12 months, while only an estimated 19.7% (95%-CI: 15.4-25.0%) of pediatricians were (corrected using TAM 1). Due to the larger number of GPs in the source population, the estimate for both groups was corrected towards the GPs’ estimate. Results were similar when using other TAM definitions. Of the 192 physicians who were consulted regarding EMF in the last year, 145 (75.5%) reported 1–4 consultations, while 25 (13.0%) reported 5–9 consultations, and 22 (11.5%) reported 10 or more. In the focus groups, physicians provided a similar assessment and reported low relevance of EMF in their everyday work. They suspected that the relevance of EMF in their everyday work is low because most people are used to EMF sources in their surroundings, because the topic is not present in the media, and because there are other more present health-related topics, e.g., COVID-19. Furthermore, pediatricians reported that parents are more worried about their children’s media and phone use than the EMF that phones emit.

In the survey, 62.6% of physicians reported poor subjective knowledge regarding EMF (Fig. 2.B) with very similar values for GPs and pediatricians as well as after correction for non-response (62.8%; 95%-CI: 57.8-67.4%). Again, using other TAM definitions did not change the results. The most frequently reported information sources were medical journals (75.7%), public service broadcasting (53.3%), and web pages of public organizations (40.8%). In the focus groups, like the online survey, most physicians reported limited knowledge regarding the potential health effects of EMF, although some did know about studies regarding EMF emitted from cell phones and cancer outcomes. There are two ways of receiving information about EMF: actively searching with the intention of getting information regarding EMF (information seeking) and passively finding information regarding EMF while accessing information sources for other reasons (information scanning). Not all physicians seek information, e.g., via a Google or PubMed search, and only get in contact with the topic when scanning their regular sources, e.g., medical journals. Physicians did communicate a need for information, especially for the current state of evidence regarding the potential health effects of EMF. Still, it was essential for them to receive information via sources they usually scan, e.g., medical journals (not necessarily highly specialized scientific journals) and newsletters, or at events they participate in, such as congresses.

## Discussion

The present study aimed to investigate GPs’ and pediatricians’ risk perception and knowledge regarding EMF as well as their relevance in everyday work by a mixed methods approach. In the online survey, about a quarter of GPs and pediatricians indicated high risk perception regarding EMF and health by agreeing that there are individuals who develop adverse health effects, especially unspecific symptoms like sleep disorders and headaches, from EMF exposure below legal limits. The specific definition of a high risk perception and the corresponding measurement method turned out to be important as, in contrast, almost half of the physicians agreed that adverse health effects from EMF are mainly psychosomatic. At the same time, the relevance of EMF in everyday work was reported to be relatively low, with about 40% of GPs and about 20% of pediatricians reporting at least one consultation during the last year, but most of them were isolated cases. Subjective knowledge regarding EMF was low as well with about 60% of physicians reporting poor knowledge. The focus groups revealed that a substantial part of GPs and pediatricians with high risk perception are physicians with poor subjective knowledge regarding EMF who cannot completely rule out EMF below legal limits as a cause of unspecific, unclear symptoms and who are therefore open to patients’ suggestions of EMF as a potential cause.

Furthermore, physicians communicated a need for information on the current state of evidence regarding the potential health effects of EMF in a format that allows them to access this information via their normal information scanning behavior, e.g., by reading medical journals. However, we observed other groups of physicians with higher risk perception regarding EMF, such as those with TAM, especially homeopathy. However, the absolute number of physicians with homeopathy training in the study population was low.

### Compatibility with other studies

Previous results on risk perception regarding EMF among GPs varied substantially: Austrian [18], Swiss [16], and German [15] studies from before 2010 reported that 77%, 61%, and between 32 and 58% of GPs, respectively, had a high risk perception regarding EMF. It is important to mention that the corresponding questionnaire items differed between the studies: the Austrian study used the negatively connoted term “electromagnetic pollution”, the Swiss study asked for adverse health effects under everyday conditions, and the German study asked for adverse health effects below legal limits. In 2017, a similar study was conducted in the Netherlands, with 62% of GPs agreeing that exposure to EMF can lead to health complaints [19]. These differences in prevalence might be partly due to differences in questionnaire items, as seen in our study, emphasizing the need for contextualization by qualitative methods. There might also be a trend towards lower risk perception due to changes in the use of technologies and other health-related topics being more present, or real differences between countries. Similar to the presented results, previous studies reported that a high proportion of GPs have poor subjective knowledge regarding EMF with values similar to ours or even higher [15–17, 19], while they reported similar [15] or somewhat higher [16] relevance during the last 12 months. Again, the specific questionnaire items differed between studies. Previous estimates were not corrected for non-response which might as well explain part of the variation. Although empirical and corrected estimates were not too different in our study, this does not necessarily translate to other studies. Pediatricians were not investigated in previous studies.

### Strengths and limitations

We conducted a mixed methods study with the possibility to contextualize survey results via focus groups and interviews. They enable individuals to communicate multilayered viewpoints, which is especially helpful when addressing a complex issue like EMF risk perception. Therefore, our study design allowed us to obtain a more comprehensive and detailed picture of physicians’ view on EMF and health than in a study relying solely on a survey. Additionally, the communication science perspective reflected in the qualitative sub-study showed clearer ways to action than the survey alone would have. We also specifically investigated the important group of pediatricians, which represents primary care for the large and potentially vulnerable group of children. To the best of our knowledge, this was the first study to assess EMF risk perception explicitly among pediatricians.

Response in the quantitative sub-study was low, which was probably due to decreasing willingness to participate in scientific studies in general [34, 35], as well as the specific investigated group of GPs and pediatricians who are usually facing a very high workload. The low response can bias prevalence estimates if sub-groups with varying prevalence also vary in their response. Therefore, we corrected our estimates using Multilevel Regression and Poststratification. The quality of the corrected estimates strongly depends on the quality of poststratification data, which in the presented study was very high since it originated from the Federal Medical Registry, mimicking the source population exactly. In addition, we used three different definitions of TAM to address differences between TAM sub-types and repeated correction once per TAM definition. However, there were no substantial differences, probably due to the small number of physicians with TAM in the source population, especially homeopathy.

Although the number of focus group participants was relatively small, theoretical saturation can be assumed because no new results were generated in later focus groups. However, no physicians who were convinced that EMF below legal limits cause adverse health effects participated in the qualitative sub-study and, in general, it can be assumed that participants had at least some interest in the topic of EMF and that completely uninterested physicians did not participate. Therefore, additional responses from these sub-groups might have been missed. In addition, due to participants being located all over Germany, the focus groups had to be conducted online. However, previous studies could not find any differences in data quality between online and offline interview settings [36]. However, the commitment to an appointment seemed lower, leading to cancellations at short notice, so some focus groups had to be conducted as in-depth interviews with a single participant. Because the same guideline was used for focus groups and in-depth interviews and as all focus group participants were encouraged to contribute equally to the discussion, the results from the in-depth interviews should be comparable with the focus group results.

## Conclusions

We investigated risk perception regarding EMF, the relevance of EMF in everyday work, and the subjective knowledge regarding EMF in German GPs and pediatricians, who play an important role in disseminating evidence-based information among the general population. Although a considerable proportion of physicians reported a high risk perception, a substantial part of these GPs and pediatricians are physicians with poor subjective knowledge regarding EMF who cannot completely rule out EMF below legal limits as a cause of unspecific, unclear symptoms, and who are therefore open to patients’ suggestions of EMF as a potential cause. Although the relevance of questions around EMF and health in physicians’ everyday work is relatively low, especially for pediatricians, there is a need for information on the current state of evidence regarding potential health effects of EMF. This information must be carried to the physicians with formats they already use in their regular information scanning behavior, such as medical journals, newsletters, or congress events.

## Electronic supplementary material

Below is the link to the electronic supplementary material.

Supplementary Material 1

Supplementary Material 2

## Data Availability

The data is not available from the authors because the use of the registry data must be approved by the Federal Ministry of Health.

## Abbreviations

CI: Confidence Interval
EMF: Electromagnetic Fields
GP: General Practitioners
ICNIRP: International Commission on Non-Ionizing Radiation Protection
MRP: Multilevel Regression and Poststratification
TAM: Additional Training in Alternative Medicine
