# Supplementary material for: Risks of electromagnetic fields from the perspective of general practitioners and pediatricians
Source: BMC Prim Care. 2025 Mar 3;26:62. doi: 10.1186/s12875-025-02762-9 (PMC11874860; doi:10.1186/s12875-025-02762-9)
Supplement: Supplementary file 1 — Supplementary Material 1 [file 12875_2025_2762_MOESM1_ESM.pdf]

Additional file 1

Questionnaire

The original questionnaire is in German (below). The questionnaire was only translated to English for presentation in this additional file. German questions were translated by one person without back-translating and should not be used without further validation.

Data on participants’ sex, age group, federal state and type of municipality in which the medical practice is located, type of physician, and additional training in alternative medicine were available in the sample we received from the Federal Medical Registry and are therefore not part of the questionnaire.

English Version

To what extent do you agree with the following statements?

“There are individuals who develop adverse health effects from electromagnetic fields below legal limits.“

Disagree				Agree
|__|	|__|	|__|	|__|	|__|

“Adverse health effects from electromagnetic fields are mainly psychosomatic.“

Disagree				Agree
|__|	|__|	|__|	|__|	|__|

In your opinion, which adverse health effects can be caused by electromagnetic fields? (Multiple answers are possible)

- ☐ Headaches
- ☐ Sleep disorders
- ☐ Nervousness / restlessness
- ☐ Difficulties concentrating
- ☐ ADHD / behavioral disorders
- ☐ Vertigo
- ☐ Tinnitus / hearing disorder
- ☐ Visual disorders
- ☐ Fatigue
- ☐ Cardiac arrhythmia
- ☐ Cancer
- ☐ Alzheimer's disease
- ☐ Other ________________

In your opinion, which sources produce electromagnetic fields that can cause adverse health effects? (Multiple answers are possible)

- ☐ Cell phones
- ☐ Cell phone base stations

☐ Cordless landline telephones
☐ Radio / TV
☐ WiFi / Bluetooth / computers
☐ Microwave
☐ Induction cooker
☐ Power lines
☐ Other _______________

Have adverse health effects due to electromagnetic fields ever come up during your consultations?

☐ Yes
☐ No

How often have health effects due to electromagnetic fields come up in your consultations during the last 12 months?

☐ 0 times
☐ 1-4 times
☐ 5-9 times
☐ 10-49 times
☐ 50-99 times
☐ ≥ 100 times

How well-informed do you perceive yourself regarding potential adverse health effects of electromagnetic fields?

Very poorly				Very well
|__|	|__|	|__|	|__|	|__|

Which sources did you use during the last 12 months when searching for information on adverse health effects of electromagnetic fields? (Multiple answers are possible)

I use …

☐ ... public service broadcasting (Radio, TV, internet services, e.g., Tagesschau or tageschau.de).
☐ … private broadcasting (Radio, TV, internet services, e.g., RTL aktuell oder RTL.de).

☐ … local or regional tabloid newspapers (e.g., Kölner Express, B.Z.).
☐ … national tabloid newspapers (e.g., Bild or bild.de).
☐ … local or regional quality newspapers (e.g., Hannoversche Allgemeine Zeitung, Osnabrücker Zeitung).
☐ … national quality newspapers or news magazines (e.g., FAZ, faz.net, Spiegel, spiegel.de).
☐ … social media posts that are shared by other users (e.g., via Facebook, Telegram).
☐ … social media posts or comments by other users (e.g., in forums, blogs, or comment sections).
☐ … so-called alternative media (e.g., Russia Today, Ken FM, Tichys Einblick, Achse des Guten, Reitschuster).
☐ … web pages of public organizations (e.g., Federal Office for Radiation Protection, World Health Organization).
☐ ... scientific databases to look for scientific articles (e.g., PubMed).
☐ … medical journals (e.g., Ärzteblatt).
☐ … other information sources: _______________
☐ … no information sources.

Regarding which aspects of health effects due to electromagnetic fields would you like more information?

____

German Version

Inwieweit stimmen Sie den folgenden Aussagen zu?

„Es gibt Personen, bei denen unter Einhaltung der gesetzlichen Grenzwerte Gesundheitsbeschwerden durch elektromagnetische Felder ausgelöst werden."

Stimme nicht zu				Stimme zu
|__|	|__|	|__|	|__|	|__|

„Gesundheitsbeschwerden durch elektromagnetische Felder sind vor allem ein psychosomatisches Problem."

Stimme nicht zu				Stimme zu
|__|	|__|	|__|	|__|	|__|

Welche Gesundheitsbeschwerden können Ihrer Einschätzung nach durch elektromagnetische Felder ausgelöst werden? (Mehrere Antworten sind möglich)

- ☐ Kopfschmerzen
- ☐ Schlafstörungen
- ☐ Nervosität / Unruhe
- ☐ Konzentrationsstörungen
- ☐ ADHS / Verhaltensauffälligkeiten
- ☐ Schwindel
- ☐ Tinnitus / Hörstörungen
- ☐ Sehstörungen
- ☐ Abgeschlagenheit / Müdigkeit
- ☐ Herzrhythmusstörungen
- ☐ Krebserkrankungen
- ☐ Alzheimer
- ☐ Sonstige ________________

Welche Quellen produzieren Ihrer Einschätzung nach elektromagnetische Felder, die Gesundheitsbeschwerden auslösen können? (Mehrere Antworten sind möglich)

- ☐ Mobiltelefone

☐ Mobilfunkbasisstationen („Handymast“)
☐ Schnurlose Festnetztelefone
☐ Radio / Fernsehen
☐ WLAN / Bluetooth / Computer
☐ Mikrowellenkochgeräte
☐ Induktionsherd
☐ Hochspannungsleitungen
☐ Sonstige _______________

Sind bei einer Ihrer Konsultationen schon einmal gesundheitliche Wirkungen elektromagnetischer Felder zur Sprache gekommen?

☐ Ja
☐ Nein

Wie oft sind während der letzten 12 Monate bei Ihren Konsultationen gesundheitliche Wirkungen elektromagnetischer Felder zur Sprache gekommen?

☐ 0 Mal
☐ 1-4 Mal
☐ 5-9 Mal
☐ 10-49 Mal
☐ 50-99 Mal
☐ ≥ 100 Mal

Wie gut fühlen Sie sich über mögliche gesundheitliche Wirkungen elektromagnetischer Felder informiert?

Sehr schlecht				Sehr gut
|__|	|__|	|__|	|__|	|__|

Welche Informationsquellen haben Sie während der vergangenen 12 Monate herangezogen, um sich über gesundheitliche Wirkungen elektromagnetischer Felder zu informieren? (Mehrere Antworten sind möglich)

Ich informiere mich …

- ☐ ... im öffentlich-rechtlichen Rundfunk (Radio, Fernsehen, Internetangebote, z. B. Tagesschau oder tageschau.de).
- ☐ … im privaten Rundfunk (Radio, Fernsehen, Internetangebote, z. B. RTL aktuell oder RTL.de).
- ☐ … in lokalen oder regionalen Boulevardzeitungen (z. B. Kölner Express, B.Z.).
- ☐ … in überregionalen Boulevardzeitungen (z. B. Bild oder bild.de).
- ☐ … in lokalen oder regionalen Qualitätszeitungen (z. B. Hannoversche Allgemeine Zeitung, Osnabrücker Zeitung).
- ☐ … in überregionalen Qualitätszeitungen oder Nachrichtenmagazinen (z. B. FAZ, faz.net, Spiegel, spiegel.de).
- ☐ ... durch Beiträge, die andere Nutzer*innen in sozialen Netzwerken teilen (z.B. Facebook, Telegram).
- ☐ … durch Beiträge oder Kommentare von Internetnutzer*innen (z. B. in Foren, Blogs, Kommentarbereichen).
- ☐ … in sogenannten alternativen Medien (z.B. Russia Today, Ken FM, Tichys Einblick, Achse des Guten, Reitschuster).
- ☐ … auf Internetseiten öffentlicher Organisationen (z.B. Bundesamt für Strahlenschutz, Weltgesundheitsorganisation).
- ☐ ... durch Recherche wissenschaftlicher Publikationen in entsprechenden Datenbanken (z.B. PubMed).
- ☐ … in medizinischen Fachzeitschriften (z. B. Ärzteblatt).
- ☐ … in sonstigen Informationsquellen, und zwar: ________________
- ☐ … nicht (ich nutze keine Informationsquellen).

Zu welchen Aspekten gesundheitlicher Wirkungen elektromagnetischer Felder wünschen Sie sich weitere Informationen?

____
