# Supplementary material for: Risks of electromagnetic fields from the perspective of general practitioners and pediatricians
Source: BMC Prim Care. 2025 Mar 3;26:62. doi: 10.1186/s12875-025-02762-9 (PMC11874860; doi:10.1186/s12875-025-02762-9)
Supplement: Supplementary file 2 — Supplementary Material 2 [file 12875_2025_2762_MOESM2_ESM.pdf]

Additional file 2

Focus group guideline

The original guideline is in German (below). The guideline was only translated to English for presentation in this additional file. German questions were translated by one person without back-translating and should not be used without further validation.

English Version

Introduction

Risk perception regarding electromagnetic fields (EMF)

From the online survey, it became clear that almost half of the physicians have already been consulted regarding health effects of EMF.

- [Risk perception] How do you assess the health risk due to EMF?
    - FOLLOW-UP PROMPT: [susceptibility] Do you think it is likely that adverse health effects are caused by EMF?
    - FOLLOW-UP PROMPT: [severity] How severe do you think adverse health effect caused by EMF are?

Subjective knowledge regarding EMF

In the online survey, your assessments of possible adverse health effects due to EMF and their sources were sometimes controversial.

- [Adverse health effects] Which adverse health effects can be caused by EMF and [EMF sources] to which sources can they be attributed?

We would now like to talk about the sources of information you use. How do you search for information on EMF?

- FOLLOW-UP PROMPT (if needed) [information seeking und scanning] Where do you come across information on EMF by chance and where do you specifically look for information on EMF?
    - Media: Which media do you use to look for information on EMF, e.g., medical journals?
    - Non-media: Which non-media sources do you use, e.g., training courses or conferences?

Need for information regarding EMF

We would now like to take a closer look at information on EMF that you are still lacking.

- [Need for information: content] Regarding which aspects of health effects due to EMF would you like more information?

    - Technology in general: Do you need more general information on technology, e.g., power lines?
    - Technology smartphone: Do you need more information on smartphones, e.g., the use of 5G?
    - Status quo / experience reports: Do you need more information on experiences in other regions of the world?
    - Effects: Do you need more information on effects of EMF, e.g., on the development of children or long-term effects?
    - Diseases and their causes: Do you need more information on the relation between EMF and specific diseases, e.g., cancer?
    - Prevention / therapy: Do you need more information on prevention and therapy?
    - Evidence / science: Do you need more information on the scientific evidence, e.g., from scientific studies on EMF effects?
- [Need for information: sources] Who should provide information on EMF (e.g., which institution/media)?
    - Who is responsible for providing and distributing information to patients?
    - Which sources are, in your opinion, not reliable?
- [Need for information: expectations] What requirements do you have for information on EMF, i.e., in what format (e.g., as a newsletter) or at what frequency (e.g., monthly or only when new findings emerge) would you like to be informed about EMF?

Conclusion

German Version

Einstieg

Risikowahrnehmung zu EMF

In unserer Online-Befragung ist deutlich geworden, dass fast die Hälfte der Ärzt*innen schon einmal in einer Konsultation auf die gesundheitlichen Wirkungen von EMF angesprochen wurden.

- [Risikowahrnehmung] Wie schätzen Sie das Risiko EMF für die Gesundheit ein?
    - NACHFRAGE: [susceptibility] Inwiefern halten Sie es für wahrscheinlich, dass Gesundheitsbeschwerden durch EMF ausgelöst werden?
    - NACHFRAGE: [severity] Für wie schwerwiegend schätzen Sie die Gesundheitsbeschwerden ein, die durch EMF ausgelöst werden?

Informations-/Kenntnisstand zu EMF

In der Online-Befragung waren Ihre Einschätzungen zu möglichen Beschwerden durch EMF und den Quellen der Beschwerden teilweise kontrovers.

- [Beschwerden] Welche Beschwerden können EMF auslösen und [EMF Quellen] auf welche Quellen lassen sich diese Beschwerden zurückführen?

Wir möchten nun noch über die von Ihnen genutzten Informationsquellen sprechen. Wie informieren Sie sich über EMF?

- NACHFRAGE (bei Bedarf) [information seeking und scanning] wo stoßen Sie zufällig auf Informationen und wo informieren Sie sich gezielt über EMF?
    - Medial: Welche Medien ziehen Sie heran, um sich über EMF zu informieren, z. B. Fachzeitschriften?
    - Non-medial: Welche nicht-medialen Quellen ziehen Sie heran, z. B. Fortbildungen oder Konferenzen?

Informationsbedürfnisse zu EMF

Wir möchten nun noch genauer darauf eingehen, welche Informationen Sie zu dem Thema vermissen.

- [Informationsbedürfnisse Inhalte] Zu welchen Aspekten gesundheitlicher Wirkungen elektromagnetischer Felder wünschen Sie sich mehr Informationen?
    - Technologie allgemein: Inwiefern haben Sie Bedarf an allgemeinen Informationen zu Technologien, z. B. Hochspannungsleitungen?
    - Technologie Smartphone: Inwiefern haben Sie Bedarf an Informationen zu Smartphones, z. B. Nutzung von 5G?
    - Status quo / Erfahrungsberichte: Inwiefern haben Sie Bedarf an Erfahrungsberichten aus anderen Regionen der Welt?
    - Wirkungen: Inwiefern haben Sie Bedarf an Informationen zu den Wirkungen EMF, z. B. auf die Entwicklung von Kindern oder Langzeitwirkungen?
    - Erkrankungen und deren Ursachen: Inwiefern haben Sie Bedarf an Informationen zum Zusammenhang von EMF und konkreten Erkrankungen, z. B. Krebserkrankungen?
    - Prävention / Therapie: Inwiefern haben Sie Bedarf an Informationen zu Präventions- oder Therapiemaßnahmen?
    - Forschungsstand / Wissenschaft: Inwiefern haben Sie Bedarf an weiteren wissenschaftlichen Erkenntnissen, z. B. Studien zur Wirkung EMF?
- [Informationsbedürfnisse Quellen] Wer sollte Informationen (z. B. welche Institution/welche Medien) zu EMF bereitstellen?
    - Wer ist für die Bereitstellung von Informationen und deren Distribution an Patient*innen verantwortlich?
    - Welche Quellen schätzen sie als nicht vertrauenswürdig ein?
- [Informationsbedürfnisse Erwartungen] Welche Anforderungen stellen Sie an Informationen zu EMF, also in welchem Format (z. B. als Newsletter) oder in welcher Frequenz (z. B. monatlich oder nur bei neuen Erkenntnissen) möchten Sie gern über EMF informiert werden?

Fazit und Abschluss
